# ﻿*Eugeniasarahchazaroi* (Myrtaceae, Myrteae), a new species from the cloud forest of Mexico

**DOI:** 10.3897/phytokeys.236.111421

**Published:** 2023-11-29

**Authors:** Antonio Francisco-Gutiérrez, Miguel Cházaro-Basáñez, Rodrigo Carral-Domínguez, Héctor Narave-Flores, Luis Islas-Tello

**Affiliations:** 1 Facultad de Biología, Universidad Veracruzana, Circuito Universitario Gonzalo Aguirre Beltrán s.n., Zona Universitaria 91000, Xalapa, Veracruz, Mexico Universidad Veracruzana Xalapa Mexico; 2 Dirección de Recursos Naturales, Secretaría de Medio Ambiente del Estado de Veracruz, Anastacio Bustamante esq. Manlio Fabio Altamirano s/n, Centro, 91000, Xalapa, Veracruz, Mexico Dirección de Recursos Naturales, Secretaría de Medio Ambiente del Estado de Veracruz Xalapa Mexico

**Keywords:** Cloud forest, Cofre de Perote, endemic species, *Eugenianaraveana*, *Umbellatae*, Veracruz

## Abstract

Following the description of *Eugenianaraveana* in 2016 from the cloud forest of the Cofre de Perote volcano, Mexico, the doubt about the existence of another unlocalized and sympatric species of *Eugenia* remained. After years of searching, the second endemic species of the Cofre de Perote volcano, *Eugeniasarahchazaroi*, is presented here. It belongs to the section Umbellatae, and is described, illustrated, and compared with *E.naraveana* and *E.coetzalensis*, recently described from Veracruz, the second state with the highest diversity of *Eugenia* in Mexico. The species is only known from the type locality and is classified in the Critically Endangered CR B1+B2(a,biii) category of the IUCN Red List conservation assessments.

## ﻿Introduction

Myrtaceae Juss. is a diverse family with ca. 6000 species distributed in tropical and subtropical regions (Lucas et al. 2019). It is classified into the subfamilies Psiloxyloideae (with two tribes) and Myrtoideae (with 17 tribes) (Wilson et al. 2005; Wilson 2010; Giaretta et al. 2019; Uc-Gala et al. 2023). From the latter, the tribe Myrteae is the most diverse within the family, with 2690 species (Stevens 2023). About 109 species of Myrtaceae are distributed in Mexico, of which 87 correspond to *Eugenia* L. (Uc-Gala et al. 2023), a monophyletic genus, one of the most hyperdiverse genera with 1218 species (Giaretta et al. 2022; POWO 2023), and the second largest genus of tree species in the world (Beech et al. 2017; Uc-Gala et al. 2023).

*Eugenia* currently circumscribes ca. 1218 species (POWO 2023). They are distributed mainly from Mexico to northern Argentina and Uruguay (including the Caribbean), with fewer species in New Caledonia, the Philippines, India, Sri Lanka, Madagascar, Mauritius, and Comores. Based on phylogenetic analyses, *Eugenia* has been classified into nine sections: *Eugenia*, *Hexachlamys*, *Phyllocalyx*, *Pilothecium*, *Pseudeugenia*, *Racemosae*, *Schizocalomyrtus*, *Speciosae*, and *Umbellatae*, which can be determined with morphological characters (Mazine et al. 2016; Giaretta et al. 2021).

This year, Uc-Gala et al. (2023) performed the most comprehensive checklist of *Eugenia* species from Mexico. They reported 87 species of *Eugenia*, 46 endemic to this country. Veracruz is the second state with the highest *Eugenia* species richness in Mexico, with 31 of them (Uc-Gala et al. 2023). Also, *Eugenia* occupies the fourth place among the richest tree genera in Mexico (Téllez et al. 2020; Uc-Gala et al. 2023). The species of *Eugenia* from Veracruz were studied in the taxonomic treatment of Myrtaceae for the Flora of Veracruz series (Sánchez-Vindas 1990). In the last decade, two new and endemic species of *Eugenia* were described from the same state, *E.naraveana* Cházaro & Franc.Gut. (Cházaro-Basáñez and Francisco-Gutiérrez 2016), and *E.coetzalensis* Durán-Esp. & Cast.-Campos (Durán-Espinosa et al. 2018).

After botanical explorations in the Cofre de Perote Volcano in Veracruz, Mexico, a new suspected species of *Eugenia* is studied here. The aims of this work are: 1) to describe a new species of *Eugenia*; 2) to compare it with the sympatric and endemic *E.naraveana*; and 3) to evaluate the conservation status of the species.

## ﻿Materials and methods

### ﻿Field work

In 2014, during the fieldwork for describing *E.naraveana*, Macario Córdova-Cortina guided the authors to one population of trees with morphological characters similar to the species collected by Miguel Cházaro-Basáñez in 1987, known as “guayabo” (guajava), but with a different vernacular name, “guayabillo” (small guajava), but it was not explored because it was decided to find the species first collected three decades ago, since both populations were considered to belong to the same taxon, the differences in fruit size being attributed to phenotypic variation. After having described and published the species (Cházaro-Basáñez and Francisco-Gutiérrez 2016), the work of the author mainly focused on the description of the species of *Agave* (Arzaba-Villalba et al. 2018, 2023) and parasitic Orobanchaceae (Francisco-Gutiérrez et al. 2019, 2023a). In July 2021, during the lockdown of the coronavirus pandemic, the fieldwork was resumed with an expedition by Miguel Cházaro-Basáñez and Héctor Narave-Flores. Finally, the species was found in the locality of Encinal II, municipality of Acajete, Veracruz, Mexico, and subsequent visits were made to collect biological and photographic material.

### ﻿Taxonomic determination

Fresh specimens of the species were collected, photographed, and dried or preserved in a solution 1:1 of ethanol and water. Measurements were made on living and preserved specimens. Voucher specimens are deposited in the cited herbaria, these cited by the acronyms following Thiers (2023). The sectional placement was determined following the sectional key provided by Mazine et al. (2016). The checklist of accepted species of *Eugenia* in Mexico (Uc-Gala et al. 2023), the taxonomic treatment of Myrtaceae in Veracruz (Sánchez-Vindas 1990), and articles of recently described species in the state (Cházaro-Basáñez and Francisco-Gutiérrez 2016; Durán-Espinosa et al. 2018) were consulted for taxonomic determination and morphological comparisons.

### ﻿Conservation assessments

Geographic coordinates were obtained in the field with a Garmin eTrex10 GPS. The data were used to estimate the geographic ranges of the extent of occurrence (EOO) and area of occupancy (AOO) with the Geospatial Conservation Assessment Tool (GeoCAT, Bachman et al. 2011) at the website http://geocat.kew.org/. The obtained values and literature on threats in the species’ distribution area were weighted to evaluate the conservation status following the Categories and Criteria of the IUCN Red List of Threatened Species (IUCN Standards and Petitions Committee 2022).

### ﻿Distribution map

The polygon of the Cofre de Perote Volcano National Park was extracted from the World Database on Protected Areas and Other Effective Area-based Conservation Measures (WDPA–WDOECM) of the United Nations Environment Programme World Conservation Monitoring Centre (UNEP-WCMC and IUCN 2021), available at https://www.protectedplanet.net/en. Digital elevation models correspond to the layer provided by WorldClim 2.1 (Fick and Hijmans 2017) with resolution of 2.5 min, and the model Continuo de Elevaciones Mexicano 3.0 of the Instituto Nacional de Estadística y Geografía (INEGI) of Mexico with resolution of 130 m, available at https://www.inegi.org.mx/app/geo2/elevacionesmex/. Map was designed in QGIS 2.15 (QGIS Development Team 2016).

## ﻿Taxonomic treatment

### ﻿ Eugenia sarahchazaroi

Taxon classificationPlantaeMyrtales

Cházaro, Franc.Gut. & J.R.Carral sp. nov.

33E3EA25-1E6C-5FDC-B1A2-D915D33580EB

urn:lsid:ipni.org:names:77331910-1

#### Diagnosis.

*Eugeniasarahchazaroi* is morphologically similar to *E.naraveana* but differs by having shorter and smaller leaves (37.3–59.7 × 14.4–21.3 mm vs. 57–116 × 22–55 mm in *E.naraveana*), reduced number of flowers per fascicle (4–6 vs. 3–16), absence of bracteoles (vs. presence), shorter pedicels (1–1.7 × 0.6–1 mm vs. 6–12 × 1–2.6 mm), smaller staminal disc (1.6–2 mm vs. 3–5 mm wide), presence of central cavity in staminal disc (vs. absence), shorter style (3.4–7 mm vs. 7.5–9.7 mm), and shorter fruits (1.4–1.8 cm vs. 1.1–4.3 cm). The species is also similar to *E.coetzalensis* but it can be distinguished by its inflorescence (axillary fascicles vs. axillary racemes in *E.coetzalensis*).

#### Type.

Mexico. Veracruz: Municipio Acajete, Paraje La Cieneguilla, cerca del Encinal II, 19.517372, -97.043692, elev. 2400 m, 01 July 2021, fl., *M. Cházaro-Basáñez & H. Narave-Flores 11226* (holotype: XAL!; isotypes: CIB!, CITRO!, ENCB!, IBUG!, MEXU!, XALU!).

#### Description.

**Figure 1.**
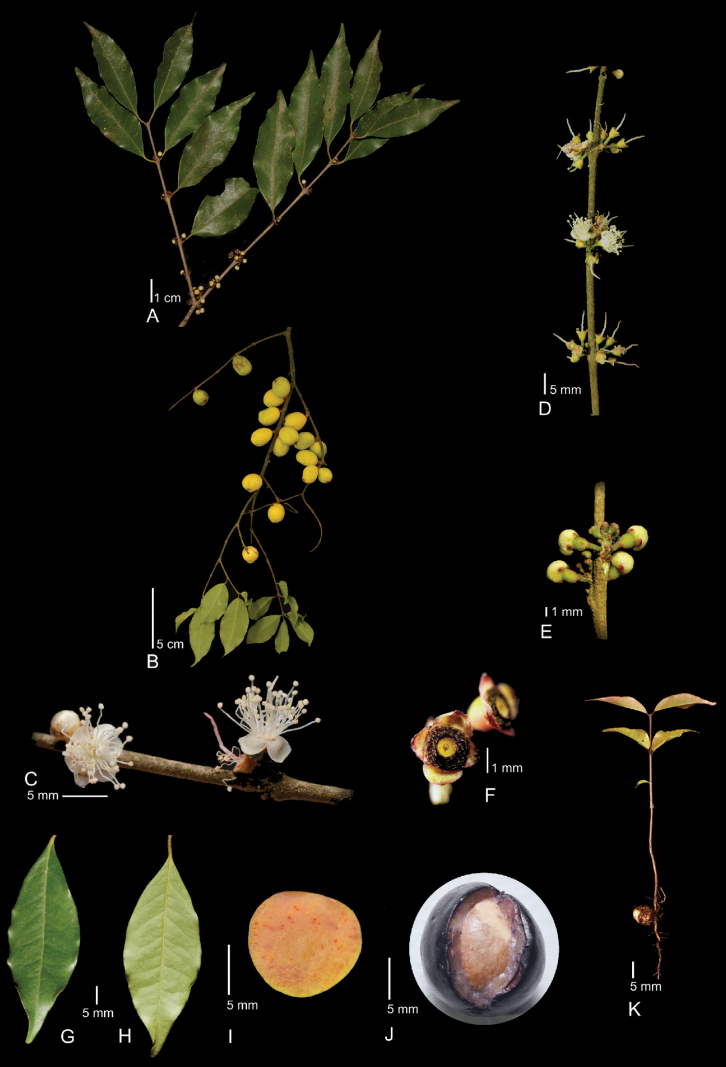
*Eugeniasarahchazaroi***A** inflorescence **B** twig with fruits**C** detail of flowers**D** fascicles**E** floral buds**F** staminal disc**G** adaxial surface of a leaf**H** abaxial surface of a leaf**I** immature fruit**J** ripe fruit, and **K** seedling. All photographs were taken by Rodrigo Carral-Domínguez and edited by Antonio Francisco-Gutiérrez.

***Tree*** 4.5–20 m tall. ***Bark*** exfoliating, the outer layer fissured and covered by lichens, the inner layer smooth and pink to reddish. Twigs terete, some covered by lichens, apical leaves paired. Internodes 15.8–21.6 mm long, 1.1–2.1 mm in diam., not exfoliating, shortly lanose to glabrescent in apical leaves. Cataphylls absent. ***Leaves*** opposite, petioles curved and adpressed, later the terminal petioles parallel to the main axis of the branches, sometimes straight and perfectly aligned one in front of the other, simulating a cross, 2.4–7.2 × 0.9–1 mm, shortly lanose. Blades 37.3–59.7 × 14.4–21.3 mm, lanceolate or elliptical, chartaceous and glossy, discolorous when dry, glabrous adaxially and abaxially, oil glands present; base cuneate, apex acuminate, 5.6–10.7 mm long, margin entire and sinuate; midvein slightly impressed adaxially, prominent abaxially, glabrous in both surfaces; secondary veins 7–14 at each side, leaving the midvein at angles of 53–76°, slightly conspicuous adaxially; one marginal vein, 0.6–1.4 mm from the margin. ***Inflorescences*** axillary fascicles, frequently 2 per node, rarely 1, 4–6 flowers each, bracts, and bracteoles absent. Pedicels of floral buds: 1.0–1.7 × 0.6–1 mm, straight to slightly curved, sometimes thickened at the base. 2-ribbed, pubescent, trichomes strigose. ***Flower buds*** ovoid to spherical, 1.1–3 mm diam., hypanthium campanulate, 1.2–2.3 × 1.5–2.2 mm, light green, shortly pubescent, trichomes simple. ***Flowers at anthesis*** with hypanthium, 1.08–1.5 × 1.4–1.9 mm, glabrous, pale green to reddish. Calyx lobes 4, free, one pair slightly less developed than the other, lobes 0.44–0.77 × 1.2–1.91 mm, widely triangular or orbicular, apex obtuse, abaxially, and adaxially glabrous, margin ciliate, trichomes 0.04–0.07 mm long, green to reddish. Petals 4, 2.4–3.2 × 2.7–2.9 mm, orbicular to elliptical, with few circular brown glands, apex widely rounded, glabrous. Staminal disc a circular ring, sometimes square with rounded corners, 1.6 mm in diameter or 1.6–2.0 mm in diameter, central cavity with no stamens inserted, 0.79–1 mm in diameter, glabrous. Stamens 31–76, deciduous, filaments 2.1–5 × 0.16–0.18 mm, glabrous; anthers 0.38–0.51 × 0.31–0.64 mm, oblong to ellipsoid, glabrous. Style 3.4–7 × 0.33–0.44 mm, glabrous, white, sometimes reddish, deciduous. Ovary locules 2, 2 ovules each. ***Fruit*** a drupe; peduncles straight or slightly curved, 4.2–4.7 × 1.9–3.8 mm; immature fruit globose to ellipsoid with some prominent and longitudinally parallel veins, 9–17.9 × 11.3–14.8 mm, smooth to reticulate, partially green, yellow or red-tinged, glabrous, not crowned at the apex with calyx lobes, pulp yellow with red granules; ripe fruit globose to ellipsoid with no veins, 14.1–18.2 × 14.7–17.2 mm, smooth, dark purple to black, glabrous; mesocarp 3.1 mm wide, salmon to dark purple; one seed per fruit, spherical to elliptical, 13.5–13.8 × 12–12.5 mm, testa smooth (Fig. 1).

#### Phenology.

The species was collected with floral buds, flowers, and mature fruits from April to July.

#### Etymology.

The specific epithet honors Sarah Magyari Cházaro-Hernández, the beloved daughter of Miguel Cházaro-Basáñez, who has accompanied him on numerous botanical expeditions. As a child, Sarah Cházaro developed an interest in botany by learning to identify several plant genera on field trips with her father (Fig. 4). This new species is the third dedicated to his children, with *Agavepaskynnellchazaroi* Arzaba, Cházaro & Franc.Gut. (Arzaba-Villalba et al. 2023), and *Valerianarudychazaroi* Cházaro, Franc.Gut. & J.R.Carral (Francisco-Gutiérrez et al. 2023b). These eponyms were the last will of Miguel Cházaro before he passed away on April 04, 2023. The obituary with a review of his life and work can be found in Francisco-Gutiérrez and Vázquez-García (2023).

#### Distribution and habitat.

The Cofre de Perote volcano, has been botanically explored since 1804 by Humboldt & Bonpland and many subsequent botanists. The mountain and its periphery house rare and new species recorded and described since the 1980’s (for a detailed review, see Cházaro et al. 2016). *Eugeniasarahchazaroi* is only known from the type locality. Other species inhabiting the cloud forest near the new species are *Quercuscorrugata* Hook., *Q.acherdophylla* Trel. (Fagaceae), *Eugenianaraveana* Cházaro & Franc.Gut., *Myrsinependuliflora* A.DC. (Primulaceae), *Ilexdiscolor* Hemsl., *Peperomiatenerrima* Schltdl. & Cham. (Piperaceae), *Lamourouxiaxalapensis* Kunth, *Pediculariscanadensis* L. (Orobanchaceae), *Oreomunneamexicana* (Standl.) J.-F.Leroy (Juglandaceae), *Echeveriasecunda* Booth ex Lindl., *E.rosea* Lindl. (Crassulaceae), *Turpiniaoccidentalis* (Sw.) G.Don (Staphyleaceae), *Ocoteadisjuncta* Lorea-Hern. (Lauraceae), *Citharexylumhidalgense* Moldenke (Verbenaceae), *Cestrumfasciculatum* Miers (Solanaceae), *Symplocoscoccinea* Bonpl. (Symplocaceae), *Oreopanaxflaccidus* Marchal (Araliaceae) and *Aporocactusflagelliformis* (L.) Lem. (Cactaceae), among many other species. It is close to the also endemic *E.naraveana*, which was only known from the type locality. Recent data allow for the expansion of its distribution, reported in the municipality of Zongolica, Veracruz (David Jimeno-Sevilla, curator of ZON herbarium, pers. comm.). An updated distribution map of *E.sarahchazaroi* and related species is provided in Fig. 3.

#### Vernacular name.

“Guayabillo” (Macario Córdova-Cortina and Héctor Narave-Flores, pers. comm.).

#### Paratypes.

Mexico. Veracruz: Municipio Acajete, Rancho de Martín Sangabriel, camino El Zapotal – El Encinal 2, km 1.52, 19.512869, -97.04118, 2320 m, 18 April 2022, fr., *R. Carral-Domínguez*, *L. Islas-Tello*, *I. Gómez-Escamilla & B. Téllez-Baños RCD-852* (IBUG!, MEXU!, XAL!); Municipio Acajete, Rancho de Martín Sangabriel, camino El Zapotal – El Encinal 2, km 1.52, 19.512974, -97.040498, 2312 m, 18 April 2023, *R. Carral-Domínguez*, *E. Marinero-Sobal* & *L. Abrajan-Cortés RCD-853* (XAL!, MEXU!); Municipio Acajete, Rancho de Martín Sangabriel, camino El Zapotal – El Encinal 2, km 1.52, 19.513025, -97.040499, 2306 m, 07 May 2023, *R. Carral-Domínguez, D. Canales-Suardíaz & A. Seedorf-Anaya RCD-854* (XAL!, MEXU!, IBUG!).

#### Conservation status.

The species has geographic ranges of Extent of Occurrence (EOO) of 0 km^2^, and Area of Occupancy of 4 km^2^. The species grows in the foothills of the Cofre de Perote Volcano, about 7.5 km from the limit of the protected area under the national park category (Fig. 3), a location that threatens it because the vicinity of the volcano has experienced constant overexploitation of forests and illegal logging since the 20^th^ century (Hoffmann 1989). Because of EOO < 100 km^2^, AOO < 10 km^2^, number of locations = 1, and continuing decline observed in the extent and quality of habitat, we evaluate the new species *E.sarahchazaroi* in the category critically endangered CR B1+B2(a,biii). This species is currently the target of conservation efforts being reproduced in the greenhouses of the Secretary of Environment and Natural Resources (SEDEMA) of Veracruz.

#### Discussion.

*Eugeniasarahchazaroi* belongs to the section Umbellatae through having bracteoles and calyx lobes not foliaceous, calyx open in the bud, and flowers 4-merous arranged in fascicles. This section has the largest species richness in *Eugenia*, with about 680 species (Mazine et al. 2016).

The species of *Eugenia* from Veracruz, Mexico, were studied in the issue of Myrtaceae of the Flora of Veracruz series (Sánchez-Vindas 1990). These species were considered for the morphological comparisons with *E.naraveana* (Cházaro-Basáñez and Francisco-Gutiérrez 2016), the most similar species. Besides, only the species *E.coetzalensis* was later described for Veracruz. Because of it, the new species presented here is compared with both.

**Figure 2.**
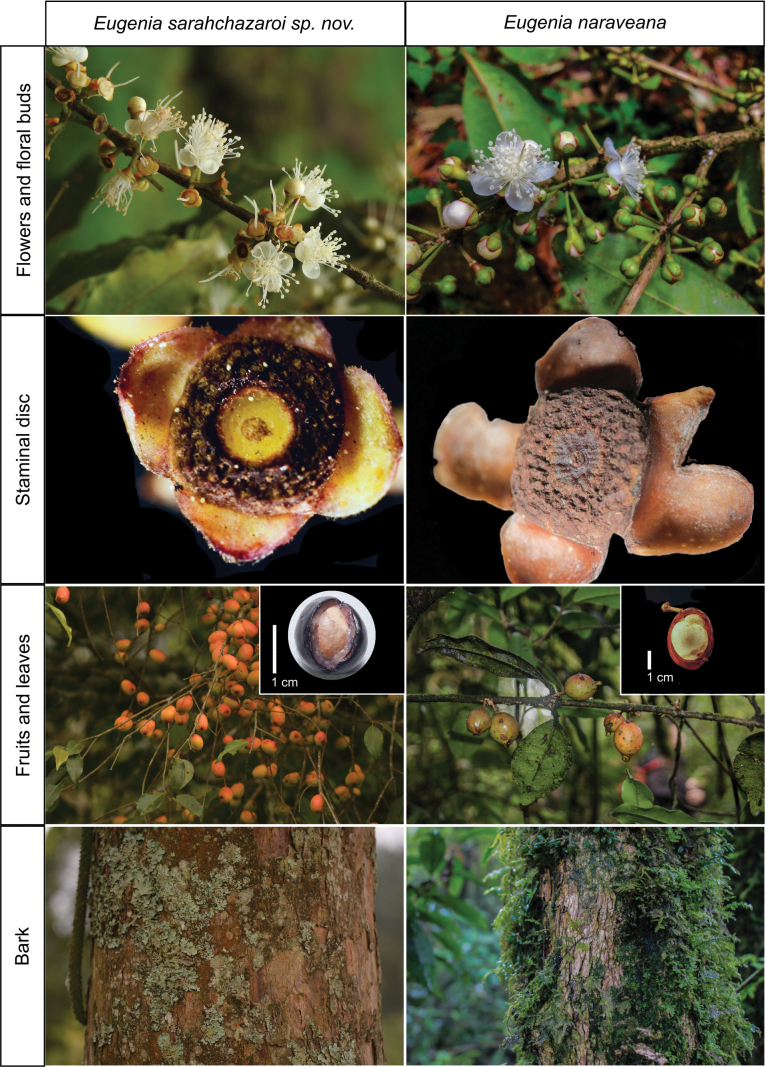
Morphological comparison between *Eugeniasarahchazaroi* and *E.naraveana*. All photographs of *E.sarahchazaroi* were taken by Rodrigo Carral-Domínguez; photographs of *E.naraveana* were taken by Antonio Francisco-Gutiérrez, except for the branch with fruits by Jose Luis Ramírez-Pacheco.

*Eugeniasarahchazaroi* is distinguished from *E.coetzalensis* mainly by the type of inflorescence (fascicle vs. racemes, respectively). The paratype of *E.coetzalensis*, *E. Guízar-N. & J.C. Echeverría 5688* (MEXU1075426) can be electronically consulted at https://datosabiertos.unam.mx/IBUNAM:MEXU:1075426. *Eugeniasarahchazaroi* is similar to *E.naraveana* but it differs in several morphological characters, which can be analyzed in the Table 1. Overall, this new species’ leaves, pedicels, hypanthium, staminal discs, and fruits are smaller than those of the *E.naraveana*. Additionally, the number of leaves and fruits are notably higher than in *E.naraveana*, as seen in photographs of Fig. 2. The staminal disc also shows a suppressed central area with no stamens where the style inserts, which is lacking in the staminal discs of *E.naraveana*. These features allow us to determine this taxon as a different species.

**Table 1.** Morphological comparison among *Eugeniasarahchazaroi* and similar species *E.naraveana* and *E.coetzalensis*.

Character	*E.sarahchazaroi*	*E.naraveana*	*E.coetzalensis*
Leaf size (mm)	37.3–59.7 × 14.4–21.3	57–116 × 22–55	23–60 × 18–33
Petiole orientation	Curved and adpressed or sometimes straight	Straight	Straight
Number of secondary veins per side	7–14	7–13	11–16
Angle of secondary veins	53–76°	60–70°	40°
Indumentum of leaf surfaces	Glabrous	Glabrous	Adpressed-strigose when newly formed, glabrescent when mature
Inflorescence and number of flowers	Axillary fascicles, frequently 2 per node, rarely 1; 4–6 flowers	Axillary fascicles, 1–2 per node; 3–16 flowers	Axillary racemes, 1 per axil, 2 per node; 2–4 flowers
Bracteoles shape	Absent	Ovate	Lanceolate
Pedicels size (mm)	1.0–1.7 × 0.6–1.0	6–12 × 1.0–2.6	8–17 × 0.4–0.5
Hypantium length (mm)	1.08–2.3	2.6–3.4	1.6–2.6
Staminal disc shape	Rounded, sometimes square with rounded corners	Quadrangular	Square
Staminal disc size	1.6 mm in diameter or 1.6–2.0 mm per side	3–5 × 3–5 mm	2 × 2 mm
Central cavity in staminal disc	Present	Absent	Unknown
Number of stamens	31–76	70–131	60–100
Style length (mm)	3.4–7	7.5–9.7	3.4–5.6
Fruit shape	Globose to ellipsoid	Subglobose	Globose
Fruit size (cm)	1.4–1.8 × 1.4–1.7	1.1–4.3 × 0.9–3.4	1.3 × 2
Fruit indumentum	Glabrous	Glabrous	Faintly strigose
Source	This study	Cházaro-Basáñez and Francisco-Gutiérrez (2016)	Durán-Espinosa et al. (2018)

**Figure 3.**
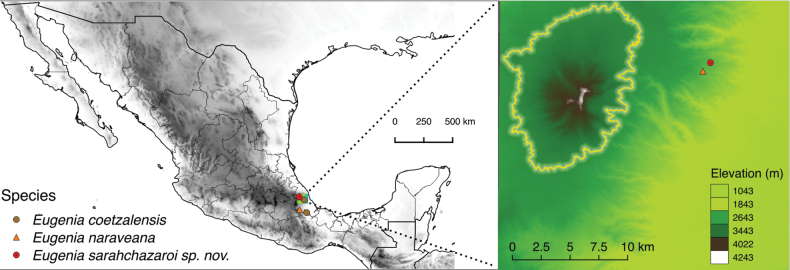
Distribution map of *Eugeniasarahchazaroi* and similar species in Mexico. The colored square corresponds to the Cofre de Perote volcano and its natural protected area under the category of National Park, delimited with the yellow line.

**Figure 4.**
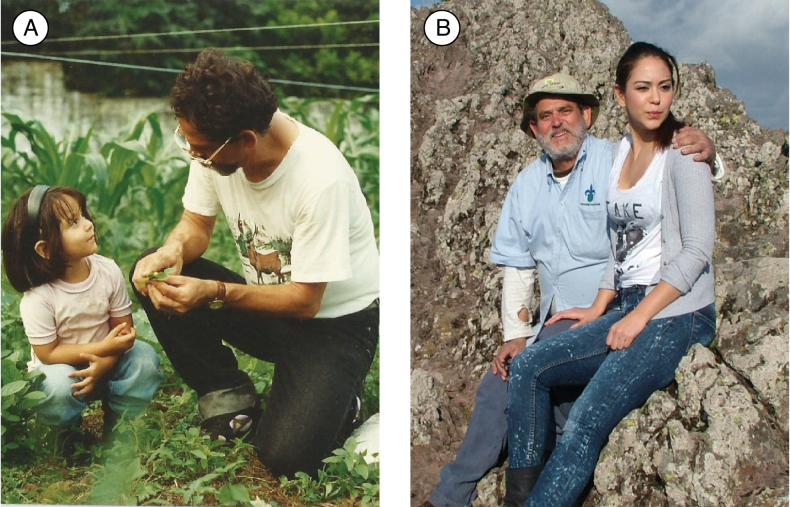
Miguel Cházaro and his beloved daughter, Sarah M. Cházaro-Hernández **A** learning her first botanical knowledge at three years old at home **B** botanical expedition in El Chico National Park, Hidalgo, Mexico, in 2018. Photographs taken by Patricia Hernández-Romero.

## Supplementary Material

XML Treatment for Eugenia sarahchazaroi

## ﻿Additional information

### Author ORCIDs

Antonio Francisco-Gutiérrez https://orcid.org/0000-0003-2013-9811

Miguel Cházaro-Basáñez https://orcid.org/0000-0003-3709-2394

Rodrigo Carral-Domínguez https://orcid.org/0000-0002-3347-5753

Héctor Narave-Flores https://orcid.org/0000-0002-3686-165X

### Data availability

All of the data that support the findings of this study are available in the main text.
